# Population-Specific Plant-To-Plant Signaling in Wild Lima Bean

**DOI:** 10.3390/plants11182320

**Published:** 2022-09-06

**Authors:** Patrick Grof-Tisza, Stéphanie Morelon, Gaylord A. Desurmont, Betty Benrey

**Affiliations:** 1 Laboratory of Evolutionary Entomology, Institute of Biology, University of Neuchâtel, 2000 Neuchatel, Switzerland; 2 European Biological Control Laboratory (EBCL), USDA-ARS, 34980 Montferrier sur Lez, France

**Keywords:** herbivore-induced plant volatiles, induced resistance, *Phaseolus lunatus*, volatile organic compounds, volatile-mediated interactions

## Abstract

The exposure to volatiles from damaged plants can increase the resistance of the neighboring plants to herbivores. Studies have demonstrated that the strength of this response depends on the level of relatedness between the interacting plants. Indeed, a field study with *Phaseolus lunatus* found that the responses to induced volatiles were population-specific; individuals exposed to damaged conspecifics from the ‘local’ population exhibited greater resistance to herbivores than those exposed to damaged conspecifics from ‘foreign’ populations. Here, we repeated this study in the laboratory by placing undamaged plants near damaged plants from either their local or a foreign population. The former plants experienced less herbivory than the latter after a subsequent challenge by a generalist herbivore. To understand the role of the volatiles underlying this observed specificity, we explored the variability in the constitutively released volatiles and volatiles released after mechanical or herbivore damage among the three tested populations of *P. lunatus*. The total volatile emissions were 5× and 10× higher from the mechanically and herbivore-damaged plants, respectively, compared to the undamaged plants. The populations differed in their relative ratios of dominant constitutive compounds, but no pattern was observed that could explain the differential responses to induced volatiles among the populations. Overall, this study confirms the population-specific volatile-mediated interactions in *P. lunatus*.

## 1. Introduction

Plants have evolved sophisticated mechanisms to sense the world around them and can selectively respond to external stimuli by altering their physiology and morphology [1,2]. One such mechanism is the ability to perceive volatile organic compounds (VOCs) indicative of risk and to respond accordingly [3]. VOCs emitted from plants contain information about their genotype and condition [4]. The blend of emitted volatiles changes when plants are attacked by herbivores [5,6,7]. These herbivore-induced plant volatiles are used as cues by undamaged tissues in the same plant or neighboring plants, priming or inducing changes that lead to increased resistance to herbivory in subsequent attacks [8,9,10,11]. While it is increasingly evident that volatile-mediated induced resistance is pervasive, the exact mechanisms underlying the ability of plants to perceive and selectively respond to VOCs cues and their evolutionary drivers are less understood [10,12].

Various factors influence the induction of defenses in plants near damaged neighbors, including the genetic relationship between the interacting plants [13]. Indeed, studies have demonstrated that plants selectively respond to cues from self- versus non-self-tissues [14,15] and kin versus non-kin [2,16,17]. More recent work has shown that plants can discriminate between cues from non-kin ‘local’ conspecifics from the same population and cues from ‘foreigner’ conspecifics from different populations [18,19]. These differential responses suggest a high level of specificity in those cues. However, our understanding of the volatile compounds constituting these cues and the mechanisms by which plants recognize them is limited. Relatively few studies have identified specific compounds that can elicit a response in neighboring plants (but see [20,21]). Experiments with sagebrush revealed that volatile bouquets of genetically related plants were more qualitatively and quantitatively similar than less related plants and demonstrated the plant’s ability to differentiate between cues depending on varying levels of relatedness, both at the intra- and inter-population levels. Although not specifically tested, this work indicated that VOCs that differentiate sagebrush chemotypes might underly the observed specificity in plant responses [18].

A recent field study on wild lima beans found that plants exposed to the VOCs of mechanically damaged plants from their own (hereafter, ‘local’) population experienced less herbivory compared to when the damaged plants were from a ‘foreign’ population [22]. Although emitted VOCs are known to vary both quantitatively and qualitatively across populations of *P. lunatus* [23], they were not analyzed for the populations studied. It is possible that the induced VOCs were unique among these populations. This interpopulation variation in induced VOCs may have underlain the specificity of responses reported, under the assumption that plants will exhibit stronger responses to more similar VOCs blends. Here, we aimed to confirm and expand on this previous work in two separate laboratory experiments. In the first confirmational experiment, we investigated whether the plant responses to induced VOCs were population-specific and tested the hypothesis that plants exposed to VOCs of plants from their local population develop higher resistance to herbivory than plants exposed to VOCs from a foreign population. In glass wind tunnels, damaged and undamaged emitter plants were placed upwind from an undamaged receiver plant from the local or a foreign population. To assess the effect of these treatments on the induced responses, the receiver plants were then challenged by a generalist chewing herbivore, *Diabrotica balteata.* We conducted a second experiment to investigate the role of volatiles underlying this observed specificity, an important component not included in earlier work. To this end, we explored the variability in the constitutively released volatiles and volatiles released from damaged plants among the tested populations of *P. lunatus.* We hypothesized that if population specificity of the volatile-mediated induced resistance was observed, it would be associated with population-level differences in VOCs.

## 2. Results

### 2.1. Volatile Exposure Experiment and Herbivore Choice Tests

**Figure 1:**
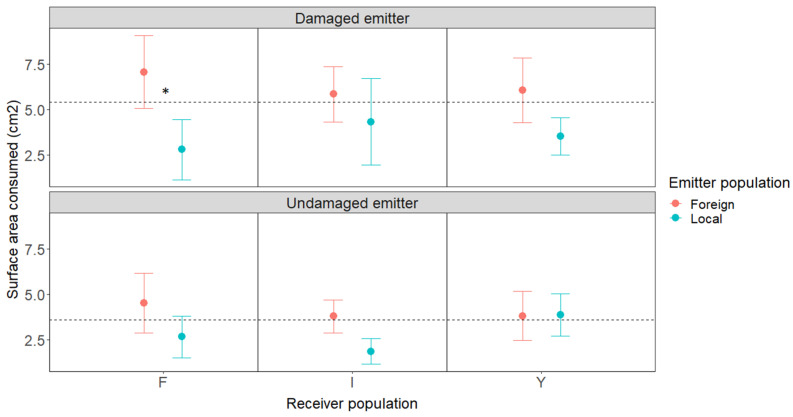
Means ± SEs from volatile exposure and herbivory experiment. F, I, and Y represent the Flores, Inka, and Yel populations, respectively. Dotted lines represent mean damage across all receiver populations for the damaged and undamaged emitter treatments. Asterisks represent significant differences (*p* < 0.05).

In alignment with the previous work, the plant responses were population-specific (Figure 1; Table 1). The receiver plants exposed to damaged emitter plants from the local populations experienced 43.5% less herbivory than plants exposed to damaged emitters from foreign populations (X^2^ = 4.29, DF = 1, *p* = 0.04). The receiver plants near undamaged neighbors from the local population experienced a non-significant 11.5% decrease in herbivory, suggesting that some plants were stressed or that low levels of damage could potentially induce defenses in neighboring plants (X^2^ = 1.44, DF = 1, *p* = 0.23). The strongest effect was associated with the Flores population; the receiver plants experienced 60.2% less damage after exposure to damage-induced VOCs from the local population relative to that from foreign populations (X^2^ = 4.17, DF = 1, *p* = 0.04). The receiver plants near damaged emitters received more damage compared to those near undamaged emitters, irrespective of the population origin of the interacting plants (Table 1).

### 2.2. Volatile Collection and GS-MS Analysis

A total of 30 VOCs with unique retention times were detected by GC-MS, 4 of which could not be identified (Table S1). The emissions of 13 compounds increased in response to the damage treatments relative to those from the undamaged controls (Table 2). Of these 13 compounds, the emissions of 10 were higher after herbivory than mechanical damage (Table 2). The total volatile emissions were 5× and 10× stronger after mechanical damage and herbivory, respectively, relative to constitutively emitted VOCs in undamaged control plants (F_2,72_ = 75.0, *p* ≤ 0.001). Although not significant, the only compound found to decrease after herbivory was methyl salicylate (*p* > 0.05). Although some compounds were differentially emitted among the three populations of *P. lunatus* (e.g., DMNT), these differences were not significant when controlling the familywise error rate (Table S1).

**Figure 2:**
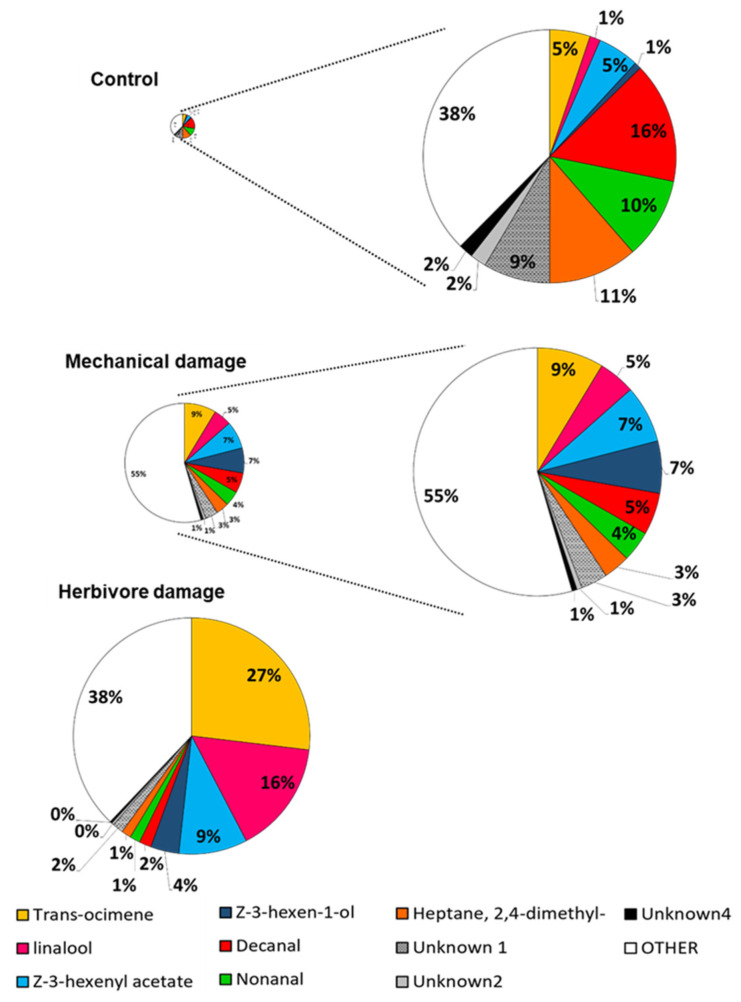
Relative proportions of VOC emissions of *P. lunatus* plants subjected to mechanical damage and herbivore damage by *D. baleata*, as well as undamaged controls. Only compounds with significant differences are depicted. Circle sizes are proportional to the average total volatile emissions of plants in each treatment.

**Figure 3:**
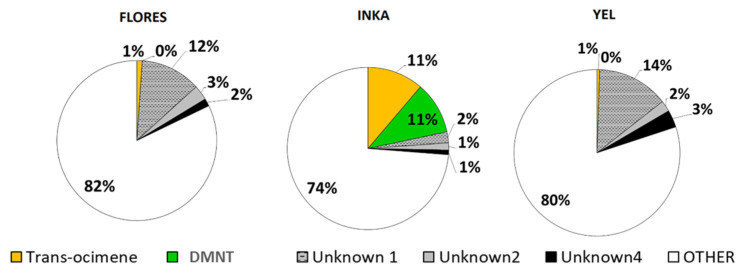
Significant variation in the relative proportions of constitutively expressed VOCs from undamaged *P. lunatus* plants. Based on these proportional differences, Flores and Yel were putatively grouped into one chemotype, with Inka representing a second chemotype.

The proportions of individual compounds constituting the total VOC blend differed between damage treatments as well as among the tested populations. The ratios of decanal; nonanal; and unknown compounds 1, 2, and 4 were lower in both damage treatments than the controls, while those of trans-β-ocimene and linalool were higher (Figure 2). The Ink population was chemically dissimilar to the Flores and Yel populations, whose constitutive VOC profiles were relatively similar to one another (Figure 3), potentially belonging to one chemotype. The Ink population had higher proportions of DMNT, trans-β-ocimene, and two unknown compounds compared to the other populations. These differences were not detected in the damage treatments (Figure S1).

### 2.3. Effects of the Geographic Relationship and Chemotype on Damage

The geographic relationships (β = −2.0 ± 0.87, R^2^ 0.47) had 27× more explanatory power than the chemotypes (β = −0.30 ± 0.84, R^2^ 0.02) between emitter and receiver plants when modeling the factors that influence the consumed surface area.

## 3. Discussion

Based on their findings of more effective communication between individuals from the same local population, Moreira et al. (2016) suggested that plants exhibit language dialects, similar to those observed in some animal species such as passerine birds. Numerous studies have demonstrated that birds can recognize regional variations (i.e., local dialects) in songs and will differently respond to individuals sharing the same dialect [24]. This analogy between plant and bird communication, however, was made without characterizing VOCs from the populations studied, which was the motivation for this current study. The results from our laboratory investigation align with this previous work as far as demonstrating that the volatile-mediated responses in *P. lunatus* were population-specific; plants exposed to mechanically damaged conspecifics from their local population experienced less herbivore damage relative to when damaged the conspecifics were from a foreign population. However, in contrast to the previous findings, the plants exposed to damage-induced VOCs were associated with higher levels of herbivory. We found that the quantity and relative proportions of emitted VOCs were strongly influenced by the type of damage *P. lunatus* received (herbivore or mechanical). However, with the compounds identified, we failed to detect population-level differences in VOC cues potentially mediating responses observed in the herbivore choice experiment. In contrast to previous work in other systems [25,26,27], the similarity of the total volatile blends in *P. lunatus* between the emitter and receiver plants did not affect the resistance response in the receiver plants. Overall, these results suggests that the chemical differences underlying population specificity are subtle, such as the minor individual compounds in the overall blend or changes in the ratios of multiple compounds, assuming the VOCs were collected during an appropriate temporal window [28,29].

Although the Flores population showed the strongest population-specific response, all three populations had a lower mean damage level after exposure to VOCs from local emitters. The idea of one population consistently emitting a stronger cue, producing deterrent VOCs that adhere to surfaces of neighboring plants, thereby providing associational resistance [30,31,32] or having stronger constitutive defenses, is not consistent with these findings. On the contrary, our results indicate that more effective signaling occurred between individuals that are likely more genetically similar.

The reason for finding that the receivers exposed to damaged plants received higher levels of damage than those exposed to undamaged plants is unclear. Our experimental design involving choice tests was devised specifically to test the effect of the relationship between plants serving as emitters and receivers (i.e., population specificity). Because the beetles could not choose among the damage treatments, this comparison was made indirectly without the ability to control for differences in the beetles’ traits (e.g., size, age) between treatments. Such indirect comparisons are common (e.g., no choice feeding assays), meaning this is not a satisfactory explanation for this unexpected finding that is indicative of induced susceptibility. It is possible that our result deviated from that reported by Moreira et al. 2016 due to our use of just one herbivore; several were thought to be attacking plants in the field, some of which may have been more sensitive to induced defenses than *D. balteata*. Despite this contrasting result, the most conclusive finding from our herbivore choice experiment is that the exposure to damaged emitters decreased the herbivore damage, but only when the emitter was from the local population. 

Volatile-mediated plant-plant interactions are well documented [33]; however, the exact mechanisms involved and the level of specificity required to elicit a response are less understood (see [13]). In some systems, mechanical damage alone is sufficient to activate defenses in neighboring plans [34]. In other systems, herbivore stimuli are needed for a response, and this will vary depending on the specific stimulus and identity of the herbivore [35]. In this study, we were focused on differences in emitted VOCs at the population-level that could account for population-dependent responses. Despite finding qualitative differences in VOCs known to be important in plant-to-plant signaling between damage treatments (e.g., indole [20], cis-jasmone [36], trans-β-ocimene [35], (*Z*)-3-Hexen-1-ol [37]), the only difference between populations detected was the proportion of constitutively expressed VOCs. Any qualitative differences may have been small and obscured by dominant compounds [38]. Although not significant, methyl salicylate, which is often emitted after attack by pathogens and non-chewing herbivores [39,40], was the only VOC to decrease in the herbivore damage treatment relative to the mechanical damage treatment. The signaling crosstalk between the salicylic acid and jasmonic acid pathways often results in reciprocal antagonism, as observed here with a chewing herbivore [41]. This finding provides confidence that our method was sensitive enough to detect the large effects of the herbivore on damage-induced VOCs beyond the physical damage they created through feeding.

A growing number of studies have demonstrated that signaling is more effective between emitter and receiver plants sharing a similar volatile blend or chemotype [25,26,27]. We found that the chemotypic relationship had very little explanatory power. A few different non-mutually exclusive reasons may explain this unexpected result. First, chemotypes often show clear discrete discontinuity of the emitted VOCs [42]. For example, the chemotypes in common thyme (*Thymus vulgaris*) have profile-dominant compounds that are not expressed in other chemotypes or are expressed to a much lesser extent. The patterns of VOC distinctness were less obvious in *P. lunatus.* In light of this, our chemotypic assignments may be less applicable or at least in the populations under study. Second, the heritability of the emitted VOCs has yet to be assessed in *P. lunatus*. The assumption of stronger genetic similarity between plants having the chemotype has not been tested in this species. It is plausible that despite the similarity in the emitted volatiles of plants assigned to the same chemotype, plants with different emitted VOCs but from the same population have more genes in common [43]. Indeed, in several plant species that exhibit chemotypic variation, chemotypes were found in geographically separated populations [42,44]. The assumption that plants of the same chemotype are more genetically related may only be valid for plants within and not between populations. More work needs to tease apart the relationship between chemotypes and genetic relatedness, as well as genetic and geographic distances.

Here, we confirmed the population-specific responses using individuals from the same populations used by Moreira and colleagues (2016). Considering this consistent pattern of induction specificity found in these two studies, a population-identifying fingerprint must be present in the volatile cue. Although we found variation in the constitutively expressed VOCs, it could not account for the differential responses observed. Two potential explanations for this are that the differences mediating population specificity are subtle, such as minor compounds emitted at low concentrations, or they depend on changes in the ratios of multiple compounds; either of these scenarios would make the detection of distinctive patterns of variation among cues more difficult. Studies of plant-insect signaling have highlighted the importance of minor compounds as well as the ratios of compounds during flower location by bees [45,46] and prey location by natural enemies [47]. More sensitive analytical equipment or more sophisticated means of detecting discriminative signatures of VOCs such as through machine learning [48,49] should be considered in future investigations. A second explanation is that the timing of the VOC collection was not appropriately delayed to detect the emission of compounds mediating plant responses. Unlike maize, which emits indole and other important signaling compounds within hours of damage by *Spodoptera* caterpillars [28,29], the emissions from damaged *Phaseolus* plants may be emitted substantially later (pers. Comm., Carla Cristina Marques Arce). Indeed, a study with cotton (*Gossypium hirsutum*) found that the induction of VOCs occurred 24–48 h after mechanical damage combined with caterpillar regurgitant [50].

## 4. Materials and Methods

### 4.1. Plant Material

The seeds were collected from three wild populations, Flores, Ink, and Yel, from the Pacific coast in Oaxaca, Mexico (see Figure 1 in [22]). The seeds were sown in pots (11 cm in height and 4 cm in diameter) and grown under greenhouse conditions (photoperiod: 16:08 D:N). The populations were separated by three meters to prevent volatile-mediated interactions, as induction by damage-induced plant volatiles is only effective within short distances (<1 m) [51]. The plants within each population had no physical contact with their neighboring plants. Fertilizer (Wuxal^®^, Duesseldorf, Germany) was used after four weeks of sowing and added once per week until the start of the experiment. When the plants had developed primary and two or three secondary leaves, they were considered large enough to use in experiments, approximately five and six weeks after planting for the first and second experiments, respectively.

### 4.2. Herbivores

The banded cucumber beetle, *Diabrotica balteata* LeConte, is a generalist leaf chewer, which is naturally present in North and South America, mostly in sub-tropical, tropical, and semi-arid climate conditions [52]. *D. balteata* is known to cause high levels of damage in crops and can cause vector diseases in *Phaseolus* spp. [53]. The individuals are commonly found feeding on the leaves of *P. lunatus* at our field sties in Mexico [22,54]. The adults were provided by Dr. Oliver Kindler from Syngenta (Stein, Switzerland). To prevent the potential habituation to *P. lunatus*, the insects were fed with Chinese cabbage (*Brassica rapa* var. pekiniensis) and maintained at an average of 25 °C with a photoperiod of 13D/11N in the quarantine facilities at the University of Neuchâtel.

### 4.3. Volatile Exposure Experiment and Herbivore Choice Tests

The plants from each population were divided in two groups: emitter plants (*n* = 40), which served as the source of VOCs; and receiver plants (*n* = 40), which were used to assess potential resistance responses. The emitter group was divided further, such that half remained undamaged to serve as controls and half were mechanically damaged using a wire brush on day one and day three of the experiment. In total, 25% of the total leaf surface area of each emitter plant within the mechanical damage treatment was wounded. Using glass wind tunnels, a receiver plant was exposed to air flowing past a closely positioned (1–3 cm) emitter plant from the local population or from a foreign population for seven days. The emitter and receiver plants had no physical contact. We chose to mechanically damage the receiver plants as opposed to exposing them to herbivores to control for the amount of damage plants received. A pilot study of the emitted volatiles expanded on in the second study presented here showed little and not significant qualitative differences in the compounds released between these two treatments. It should be noted that this replicated experiment was only conducted once.

**Figure 4:**
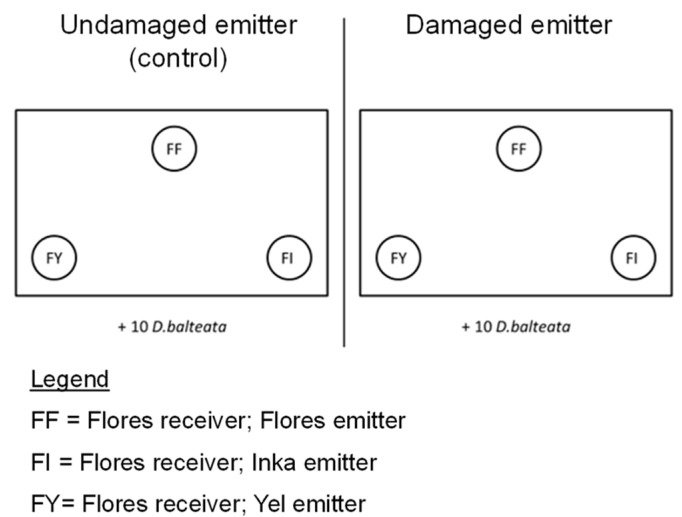
Design of the choice test feeding assay. For each treatment (damaged, *n* = 15; undamaged, *n* = 15), three receiver plants from the same population were put in a cage alone with 10 *D. balteata* adults for 24 h. Five replicate cages for each combination were used.

A choice test was used to assess the effect of the geographic relationship (i.e., population) between the emitter and receiver plants on the induced resistance. The choice test consisted of placing three receiver plants from the same population in a large cage (30 × 30 × 30 cm; Vermandel products, Hulst, The Netherlands) with 10 *D. balteata* adults (Figure 1). All combinations of receiver and emitter plants from the three populations were tested. The receiver populations were tested separately from one another as a means to control for underlying preference differences by *D. baleata*. The exposure to VOCs from damaged plants can prime and directly induce VOCs in *P. lunatus* [8,55,56]. Thus, to ensure that the receiver plants paired with undamaged neighbors served as uninduced controls, we also separately tested the receiver plants exposed to damaged (*n* = 15) and undamaged (*n* = 15) emitter plants (Figure 4).

### 4.4. Volatile Collection and GS-MS Analysis

Thirty plants from the Flores and Inka groups and 24 plants from the Yel group were assigned to three treatments: damaged mechanically, damaged by the herbivore *D. balteata*, and undamaged controls. A wire brush was used to damage the surfaces of primary and secondary leaves in the mechanical damage treatment. The plants were then placed into glass bottles (diameter: 6 cm; length: 35 cm). For the herbivore damage treatment, three *D. balteata* individuals were positioned onto leaves and then the plants were placed into glass bottles. The bottles were incubated for 24 h at room temperature under a light a 16:8 L/D photoperiod. After 24 h, the insects were removed from bottles in the herbivore damage treatment and the plants in the mechanical damage treatment were damaged a second time. In total, the wire brush was pressed on 25% of the total leaf surface of the plant. The volatiles were drawn from the bottles at a flow rate of 400 mL/min^−1^ and collected in Super Q filters for six hours. The collected volatiles were subsequently extracted with 100 μL of pure dichloromethane and stored in a −80 °C freezer.

This single experiment was staggered over four days. The volatiles were also collected in empty bottles and in bottles containing only *D. balteata* to detect the presence of possible contaminants. At the termination of the experiment, the leaves from each plant were collected and scanned and analyzed as described above to quantify the herbivore damage. The fresh biomass of each plant was also recorded.

The VOCs were analyzed with an Agilent 6890 gas chromatograph with a flame ionization detector. Here, a 2 µL aliquot of each sample was injected in the pulsed splitless mode onto a non-polar column. Helium was used as the carrier gas. 1-Bromodecane was used as the internal standard for calibration. After injection, the temperature was maintained at 40 °C for 3 min, then increased to 100 °C at 8 °C/min and to 220 °C at 5 °C/min. The quantities of the major components of the blends were estimated based on the peak areas of the compounds compared to the peak areas of the internal standards consisting of 20 ng μL^−1^ n-octane and nonyl-acetate. The compounds were identified by comparing the spectra obtained from the samples with those from a reference database (NIST mass spectral library).

### 4.5. Statistical Analysis

All statistical analyses were conducted using R and R studio versions 4.0.3 (herbivore analysis) and 3.2.5 (volatile analysis). The linear mixed effect models (package glmmTMB) [57] were used to explore the relationship between the population origin of the receiver plants and emitter plants further categorized as ‘local’ and ‘foreign’ with respect to the population of the receiver plant on the consumed leaf surface area. Cages nested within the data were used as random variables. The model performance was assessed using the Dharma package [58]. Because the herbivores were not free to choose among the receiver plants from all populations and those exposed to undamaged and damaged emitters, caution must be exercised when directly comparing between populations and damage treatments. Therefore, in addition to this global model, we specifically compared the effect of the population origin (local versus foreign) on the surface area consumed for each receiver population in the undamaged and damaged treatments separately using the same model structure as described above.

An analysis of covariance (ANCOVA) was used to assess the effects of the population and damage treatment on the quantity (ng) and ratio (% of volatiles produced by the plant) of the individual compounds, as well as the total volatile emission. The plant biomass was used as a covariate. All possible interactive effects were included. The total volatile emission was log-transformed and the individual emissions for each compound were bootstrapped by 1000 to conform to the model assumptions. A Bonferroni correction was applied to account for the large number of parallel comparisons (for α = 0.05/30 = 0.0017). Post hoc Tukey tests were used to assess the significance of the levels within significant factors. The plant size and total damage by herbivores were compared between all populations to ensure there was no underlying preference differences for *D. Balteata*.

### 4.6. Effects of the Geographic Relationship and Chemotype on the Damage

After the GS-MS analysis, we identified two putative chemotypes based on the relative proportions of constitutively expressed VOCs, specifically the proportions of trans-β-ocimene, DMNT, and two unknown compounds. We conducted a post hoc analysis to compare the explanatory power of the chemotype and population (i.e., foreign vs. local) when modeling the surface area consumed using the same model structure described for the volatile exposure experiment.

## 5. Conclusions

*P. lunatus* showed population specificity in its responses to volatile cues, with stronger induced responses when those cues emanated from conspecifics from the local population. While this is suggestive of population dialects, no chemicals mediating population specificity were detected. Overall, this work suggests that chemical mediators in plant-to-plant signaling may be subtle, similar to what has been described in plant–insect signaling, or may be delayed with their emission, occurring more than six hours after the secondary damage. Studies such as the one presented here add to the growing list of those cementing the phenomenon of volatile-mediated plant–plant interactions. The continued challenge in the field is the identification of compounds either subtle or otherwise that are sufficient to elicit responses in plants, especially those involved with herbivore resistance. Considering the great implications of inducible defenses on plant survival and yield for natural and agricultural systems alike, such discoveries could guide conservation management and agricultural practices in a quickly changing world.

## Figures and Tables

**Table 1:** Results from a GLMM of an herbivory assay with *D. balteata* and *P. lunatus* plants from three populations exposed to damaged (Dam.) or undamaged (Und.) emitter plants (treatment) from local (Loc.) or foreign (For.) populations.

Factor	*X^2^*	DF	*p*
Receiver pop.	0.248	2	0.883
Emitter (Loc. vs. For.)	0.578	2	0.016
Treatment (Dam. Vs. Und.)	4.890	1	0.027

**Table 2:** Means ± SEs of individual compound emissions (ng/6 h/plant) averaged over three populations of *P. Lunatus* (Flores, Inka, Yel) from three damage treatments, including an undamaged control. Only compounds that were found to be significantly different from the undamaged treatment are included. Bolded compounds indicate those that were significantly higher in the herbivore damage than mechanical damage treatment. Tukey HSD tests were used to assess pairwise significance.

Volatile Compound	Undamaged	Mechanical Damage	Herbivore Damage	F Test	*p* Value
**Trans-β-ocimene**	1.61 ± 0.78	13.57 ± 4.59	88.82 ± 9.93	53.51	<0.001
**DMNT** ^1^	1.36 ± 0.83	21.23 ± 7.3	54.4 ± 11.3	26.88	<0.001
**Linalool**	0.43 ± 0.32	7.52 ± 1.84	51.09 ± 8.98	22	<0.001
**Z-3-hexenyl acetate**	1.65 ± 0.97	11.76 ± 2.54	30.96 ± 4.75	27.46	<0.001
Indole	1.76 ± 0.58	13.24 ± 2.72	15.31 ± 2.66	13.56	<0.001
Z-3-hexen-1-ol	0.27 ± 0.15	10.66 ± 2.32	12.83 ± 1.96	19.27	<0.001
**TMTT** ^2^	0.33 ± 0.19	3.44 ± 0.9	9.61 ± 1.82	6.22	<0.001
Methyl salicylate	0.72 ± 0.22	6.98 ± 2.16	4.11 ± 1.27	9.58	<0.001
**Trans-caryophyllene**	0.11 ± 0.05	1.7 ± 0.64	3.92 ± 0.85	9.13	<0.001
**Cis-jasmone**	0.21 ± 0.11	1.85 ± 0.59	3.8 ± 0.87	8.29	<0.001
**Cis-3-hexenyl isovalerate**	0.28 ± 0.13	1.4 ± 0.51	3.24 ± 0.51	20.48	<0.001
**Trans-nerolidol**	0.03 ± 0.03	1.29 ± 0.5	2.71 ± 0.33	16.02	<0.001
**Cis-β-ocimene**	0.33 ± 0.19	1.36 ± 0.43	2.66 ± 0.34	12.92	<0.001

^1^ (E)-4,8-Dimethyl-1,3,7-nonatriene. ^2^ (E, E)-4,8,12-Trimethyl-1,3,12-trimethyl-1,3,7,11-tridecatetraene.

## Supplementary Materials

The following are available online at https://www.mdpi.com/article/10.3390/plants11182320/s1: Figure S1: Significant variation in the relative proportions of constitutively expressed volatile organic compounds from damaged *P. lunatus* plants; Table S1: Emissions (ng/6 hours/plant) and unique retention times for all compound for the three damage treatments, including the undamaged control, for all three *Phaseolus lunatus* populations.
