# Supplementary material for: Population-Specific Plant-To-Plant Signaling in Wild Lima Bean
Source: Plants (Basel). 2022 Sep 6;11(18):2320. doi: 10.3390/plants11182320 (PMC9503452; doi:10.3390/plants11182320)
Supplement: Supplementary file 1 [file plants-11-02320-s001.zip › plants-1821673-supplementary.pdf]

Supplemental material.

Table S1. Emissions (ng/6 hours/ plant) and unique retention times for all compound for the three damage treatments, including the undamaged control, for all three *Phaseolus lunatus* populations.

Compound	Retention time	Flores			Inka			Yel		
		Control	Mechanical damage	Herbivore damage	Control	Mechanical damage	Herbivore damage	Control	Mechanical damage	Herbivore damage
Z-3-hexenal	5.2	0.85 ± 0.85	1.82 ± 1.44	3.43 ± 1.34	0.00 ± 0.00	4.22 ± 1.95	4.55 ± 2.01	0.00 ± 0.00	3.28 ± 2.35	5.56 ± 2.21
2,4-dimethyl-heptane	5.7	2.99 ± 0.69	7.28 ± 1.53	3.44 ± 1.13	2.46 ± 0.76	3.93 ± 1.06	5.17 ± 1.22	5.57 ± 1.16	4.65 ± 1.21	4.62 ± 1.03
4-hydroxy-4-methyl-2-pentanone	6.2	1.38 ± 0.68	2.04 ± 0.63	1.42 ± 0.77	0.44 ± 0.36	1.56 ± 0.18	2.54 ± 1.93	1.24 ± 0.33	1.53 ± 0.56	2.65 ± 0.73
Z-3-hexen-1-ol	6.4	0.06 ± 0.06	5.72 ± 1.09	9.88 ± 1.81	0.56 ± 0.39	14.76 ± 3.26	21.11 ± 3.76	0.15 ± 0.15	12.73 ± 6.52	6.16 ± 2.00
E-2-hexenal	6.5	0.12 ± 0.08	0.93 ± 0.33	2.47 ± 0.71	0.05 ± 0.05	0.72 ± 0.31	3.12 ± 0.70	0.47 ± 0.18	1.94 ± 1.63	1.53 ± 0.66
Heptanal	7.6	0.07 ± 0.07	0.67 ± 0.36	0.00 ± 0.00	0.00 ± 0.00	0.14 ± 0.14	0.00 ± 0.00	0.15 ± 0.15	0.29 ± 0.19	0.87 ± 0.34
Benzaldehyde	8.9	0.13 ± 0.11	2.51 ± 1.24	0.66 ± 0.27	0.07 ± 0.07	0.69 ± 0.35	1.90 ± 0.33	0.64 ± 0.64	0.46 ± 0.33	2.34 ± 0.82
2,4,6-trimethyl-pyridine	9.6	0.34 ± 0.34	0.81 ± 0.42	0.89 ± 0.52	0.02 ± 0.02	0.24 ± 0.16	1.09 ± 0.43	0.07 ± 0.07	0.42 ± 0.29	2.74 ± 1.65
Octanal	9.9	0.78 ± 0.32	2.29 ± 0.69	0.73 ± 0.26	0.33 ± 0.18	1.08 ± 0.41	0.70 ± 0.23	0.77 ± 0.39	1.23 ± 0.40	1.83 ± 0.55
Z-3-hexenyl acetate	10.0	0.28 ± 0.15	10.51 ± 3.87	28.00 ± 7.49	3.85 ± 2.55	16.11 ± 5.10	48.11 ± 8.02	0.44 ± 0.22	8.97 ± 4.55	13.22 ± 3.49
R-Limonene	10.4	1.73 ± 0.57	9.12 ± 4.13	1.65 ± 0.31	2.19 ± 1.20	5.78 ± 2.92	4.84 ± 2.06	1.89 ± 0.86	1.07 ± 0.44	3.96 ± 1.85
Cis-β-ocimene	10.6	0.19 ± 0.13	2.19 ± 1.05	2.59 ± 0.39	0.07 ± 0.05	0.87 ± 0.18	2.07 ± 0.32	0.80 ± 0.63	0.82 ± 0.37	3.48 ± 0.97
Unknown.1	10.7	3.23 ± 1.02	7.31 ± 2.32	3.56 ± 1.49	0.77 ± 0.69	4.24 ± 0.99	4.92 ± 2.24	4.37 ± 1.29	4.26 ± 1.64	6.65 ± 2.30
Trans-β-ocimene	10.8	0.29 ± 0.23	6.99 ± 2.16	94.40 ± 18.46	3.97 ± 1.94	39.09 ± 13.82	85.80 ± 13.92	0.17 ± 0.17	10.77 ± 4.42	85.62 ± 21.53
Linalool	11.9	0.00 ± 0.00	5.14 ± 1.09	53.65 ± 13.10	0.84 ± 0.84	11.00 ± 4.22	53.74 ± 11.52	0.39 ± 0.28	7.03 ± 4.10	44.57 ± 24.30
Nonanal	12.0	3.40 ± 0.73	8.88 ± 2.45	3.97 ± 0.42	2.33 ± 0.75	4.45 ± 0.80	4.21 ± 0.38	4.22 ± 1.19	4.70 ± 0.65	5.96 ± 1.51
DMNT[1]	12.3	0.00 ± 0.00	3.75 ± 1.68	38.17 ± 12.11	3.67 ± 2.12	42.35 ± 18.55	87.15 ± 25.61	0.00 ± 0.00	21.97 ± 12.32	33.75 ± 11.62
2-(2-butoxyethoxy)-ethanol	13.9	0.32 ± 0.22	17.34 ± 10.58	0.32 ± 0.22	5.26 ± 3.95	29.41 ± 24.72	11.80 ± 8.42	0.00 ± 0.00	0.00 ± 0.00	3.82 ± 3.43
Methyl salicylate	14.1	0.51 ± 0.34	4.74 ± 1.54	1.20 ± 0.49	0.97 ± 0.39	13.91 ± 6.20	7.86 ± 3.11	0.65 ± 0.44	2.84 ± 1.34	3.08 ± 1.30
Decanal	14.3	5.48 ± 1.55	10.91 ± 2.64	3.67 ± 0.42	3.46 ± 1.31	7.27 ± 1.40	4.83 ± 0.78	5.77 ± 1.70	7.00 ± 1.23	8.46 ± 2.96
Cis-3-hexenyl isovalerate	14.9	0.22 ± 0.22	0.92 ± 0.47	3.43 ± 1.13	0.40 ± 0.26	2.04 ± 1.37	3.88 ± 0.64	0.20 ± 0.20	1.36 ± 0.85	2.20 ± 0.71
Indole	16.4	2.48 ± 1.48	15.03 ± 4.40	12.86 ± 4.01	1.35 ± 0.70	15.54 ± 6.30	21.69 ± 5.66	1.46 ± 0.68	8.70 ± 3.30	10.39 ± 2.51
Unknown3	18.4	0.00 ± 0.00	2.49 ± 1.57	0.80 ± 0.60	0.00 ± 0.00	0.67 ± 0.36	1.31 ± 0.87	0.00 ± 0.00	1.00 ± 0.69	0.00 ± 0.00
Cis-jasmone	18.9	0.17 ± 0.12	3.14 ± 1.17	3.13 ± 0.90	0.35 ± 0.27	1.66 ± 1.06	5.77 ± 2.15	0.10 ± 0.10	0.44 ± 0.31	2.18 ± 0.63
Trans-caryophyllene	19.4	0.22 ± 0.11	2.57 ± 1.51	5.52 ± 1.79	0.09 ± 0.06	1.15 ± 0.53	4.28 ± 1.31	0.00 ± 0.00	1.15 ± 0.76	1.46 ± 0.66
Unknown2	21.2	0.80 ± 0.11	1.37 ± 0.36	0.92 ± 0.21	0.50 ± 0.14	0.83 ± 0.08	1.24 ± 0.23	0.67 ± 0.03	0.91 ± 0.08	0.87 ± 0.10
α-Farnesene	21.4	0.00 ± 0.00	0.72 ± 0.28	4.50 ± 2.32	0.07 ± 0.07	0.09 ± 0.06	0.85 ± 0.47	0.00 ± 0.00	0.18 ± 0.09	2.10 ± 1.25
Unknown4	22.1	0.41 ± 0.14	1.05 ± 0.31	0.58 ± 0.18	0.33 ± 0.14	1.26 ± 0.66	1.14 ± 0.21	1.06 ± 0.22	0.68 ± 0.19	1.05 ± 0.30
Trans-nerolidol	22.7	0.00 ± 0.00	2.07 ± 1.25	2.56 ± 0.46	0.08 ± 0.08	1.02 ± 0.37	3.89 ± 0.50	0.00 ± 0.00	0.59 ± 0.30	1.42 ± 0.47
TMTT[2]	23.0	0.05 ± 0.05	1.17 ± 0.40	8.05 ± 2.31	0.74 ± 0.50	6.30 ± 2.02	14.02 ± 4.11	0.15 ± 0.10	3.40 ± 1.72	6.04 ± 1.85
TOTAL	100.0	26.47 ± 5.80	141.48 ± 29.55	296.48 ± 31.91	35.19 ± 12.02	217.89 ± 52.78	413.53 ± 50.26	31.39 ± 4.87	114.35 ± 39.94	268.57 ± 53.38

[1] (E)-4,8-dimethyl-1,3,7-nonatriene

[2] (E, E)-4,8,12-trimethyl-1,3,12-trimethyl-1,3,7,11-tridecatetraene

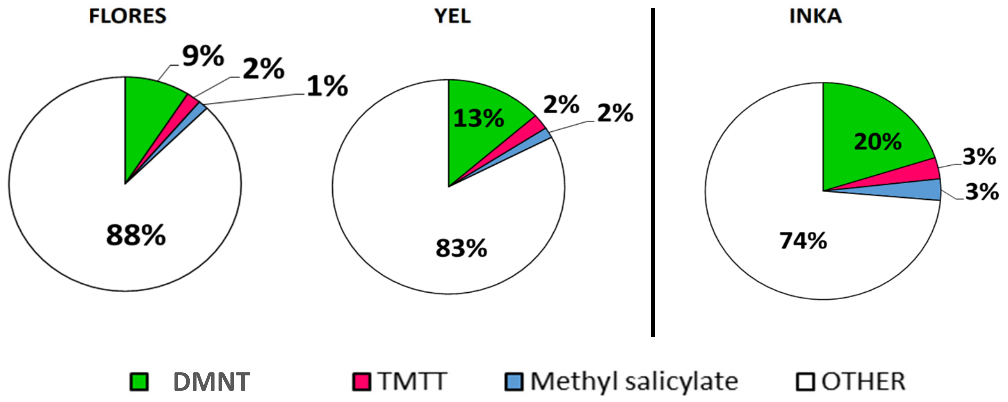

Figure S1. Significant variation in the relative proportions of constitutively expressed volatile organic compounds from damaged *P. lunatus* plants. DMNT and TMTT represent (E)-4,8-dimethyl-1,3,7-nonatriene, (E, E)-4,8,12-trimethyl-1,3,12-trimethyl-1,3,7,11-tridecatetraene. respectively.
